# Genetic diversity of group A rotavirus in acute gastroenteritis outpatients in Shanghai from 2017 to 2018

**DOI:** 10.1186/s12879-020-05279-x

**Published:** 2020-08-12

**Authors:** Xiaozhou Kuang, Xiaohuan Gong, Xi Zhang, Hao Pan, Zheng Teng

**Affiliations:** 1 Shanghai Municipal Center for Disease Control and Prevention, 1380 Zhongshan Road (west), Shanghai, 200336 China; 2 Shanghai Institutes of Preventive Medicine, 1380 Zhongshan Road (west), Shanghai, 200336 China

**Keywords:** Group A rotavirus, Acute gastroenteritis, Outpatients, Genotype, Antibiotics, Fecal shedding

## Abstract

**Background:**

Group A Rotavirus (RVA), despite being an important pathogen in hospitalized children, is less studied in pediatric outpatients, and even rarely investigated in adults. This study aims to understand the genetic diversity of RVA in outpatients across all age groups in Shanghai, and thus providing a molecular basis for vaccine implementation and evaluation.

**Methods:**

Stool samples were first screened by Real-time Reverse Transcription Polymerase Chain Reaction (rRT-PCR). RVA genotyping was performed through the amplification of partial VP7 and VP4 gene. Strains of interest were further sequenced and analyzed using MEGA 6.0.

**Results:**

Four thousand nine hundred one samples were collected, from which 7.61% (373 cases) were screened positive for RVA. RVA prevalence was higher in children (9.30%) than in adults (7.21%) (χ^2^ = 4.72, *P* < 0.05). 9.38% RVA positive cases had taken antibiotics before hospital visit while 49.60% had been prescribed antibiotics afterwards. RVA displayed a strong seasonality in both adults and children with a shared commonality in genotype repertoire, where G9P[8] was the most prevalent strain (67.96%) followed by G3P[8] (15.49%) and G1P[8] (12.32%). Meanwhile the first local case of fecal shedding of the G10P[15] vaccine strain was also discovered.

**Conclusions:**

While the prevalence of rotavirus is highest during cold seasons, it is revealed for the first time that G9P[8] is the predominant genotype in both adults and pediatric outpatients. Clinically, higher occurrence of nausea or vomiting was observed in RVA positive cases. Antibiotic overuse was implicated in both non-clinical and clinical settings. The finding emphasizes the importance of RVA genotyping in surveillance as it provides the basis for new vaccine application as well as a baseline for future vaccine efficacy evaluation.

## Background

Rotavirus is an important pathogen in viral gastroenteritis, especially in children. According to WHO, rotavirus still caused 215,000 estimated deaths in < 5 years old children globally by 2013 despite various vaccination efforts [1]. In 2016, a global disease burden study covering 195 countries has also reported that rotavirus was the leading etiology for diarrhea mortality among children under 5 years and among all ages [2]. In China, rotavirus caused around 40% and 30% of diarrhea-related hospitalizations and outpatient visits, respectively, among children aged < 5 years [3].

Among three major rotavirus groups that can infect human and animals (group A, B, C), group A rotavirus (RVA) is the most important group in terms of epidemiological and clinical impact in human population [4]. Our local previous study from 2012 to 2016 has also shown that RVA accounted for 97.3% of all rotavirus infections in adult acute diarrhea outpatients [5]. Moreover, of all viral pathogens that cause acute diarrhea, only RVA is preventable through vaccination.

RVA is classified by a binary classification system based on immunological reactions and the structure of two most external proteins VP7 (or G genotype) and VP4 protein genes (or P genotype), which binds to their neutralizing antibodies independently [4]. Up to now, at least 27 G genotypes and 37 P genotypes of RVA have been reported in humans and animals [4]. The predominant RVA genotype circulating in China in recent years is G9P[8] [6], which is different from the prevalent genotypes such as G3P[8] and G1P[8] in the neighboring countries in South East Asia [7]. In Shanghai, RVA genotype distribution has only been investigated in limited sentinel hospitals, involving < 5 yrs. inpatients. The predominant genotype in this population was G9P[8] [8]. However, RVA genotype diversity in outpatients involving all age groups remains to be discerned. Therefore, it is the very first time in local history that RVA profiles in both pediatric and adults outpatients involving large scale surveillance programme were ever investigated.

Regarding RVA related disease prevention In China, the Lanzhou lamb rotavirus (LLR) vaccine, despite not being included in the national mandatory vaccination programme, was the only available option in the last two decades until very recently. At the end of 2018, RotaTeq vaccine has been launched (though neither was it on the mandatory vaccine list) with a more promising efficacy by its decade long use around the world [9].

This study aims to gain insight into the genetic diversity of RVA in both children and adults in providing the basis for measuring vaccine efficacy in the near future.

## Methods

### Case definition, sentinel hospital recruitment, sampling method, stool sample process and RNA extraction

This study was conducted as one of the major parts of the Comprehensive Surveillance Programme of Diarrheal Diseases in Shanghai. The case definition, as well as procedures such as sentinel hospital recruitment, sampling method, stool sample process and RNA extraction was outlined in previous studies [10, 11].

### Real-time reverse transcription polymerase chain reaction (rRT-PCR) screening

All specimens were double screened for RVA by rRT-PCR using commercially available kits (Shanghai Zhijiang Biotechnology Co. Ltd. (a.k.a. Liferiver Bio-Tech), and Jiangsu Shuoshi Biotechnology Co., Ltd. (a.k.a. BioPerfectus Technologies)) according to the manufacturer’s instructions.

### RVA genotyping

For samples that were positive in both rRT-PCR kits, VP4 and VP7 regions were amplified by nested PCR using reagents and primers detailed in literature [12–15]. Similarly, genotypes were determined according to their respective product size by capillary gel electrophoresis described previously [12–15]. Strains of interest were further sequenced, and the resultant sequences spliced using the same methods from earlier studies [11, 16]. These sequences were then classified by RVA online genotyping tool (http://rotac.regatools.be, RotaC, Leuven, Belgium) [17]. Sequences representative of the main variants of strains which are of focus in this study were deposited in GenBank (accession numbers MN816183–202; MN833128–138).

### Phylogenetic analysis

For strains of interest, phylogenetic trees were constructed in MEGA version 6.0 by applying the maximum likelihood method, using the substitution models suggested by the MEGA6 model test for each tree [18]. Tree robustness was determined by bootstrapping using 1000 pseudo replicates [19].

### Statistical analysis

All calculations were conducted using Microsoft Excel 2010 and SPSS software v16.0 (IBM, USA), where Cochren Mantel-Haenszel Chi square test and the Fisher’s exact with two-tailed method were used to determine statistical significance with *P* < 0.05.

## Results

### Overall demographic analysis on RVA positive detection rate

From January 2017 to December 2018, a total of 4901 stool samples with epidemiological information were collected from diarrhea outpatients, including 2611 samples from 2017, and 2290 samples from 2018. A total of 373 samples (7.61%) were positive for RVA by rRT-PCR (Table 1). The overall RVA positive rate in children was significantly higher than that in adults (χ^2^ = 4.72, *P* < 0.05).

**Table 1:** RVA positive rates by rRT-PCR screening in adult and pediatric outpatients in 2017–2018

	Adults				Children			
Year	No. of cases	No. of RVA POS	POS rate (%)	χ^2^ (*P*-value)	No. of cases	No. of RVA POS	POS rate (%)	χ^2^ (*P*-value)
2017	2115	155	7.33	0.09 (0.76)	496	39	7.86	2.60 (0.11)
2018	1851	131	7.08		439	48	10.93	
Total	3966	286	7.21		935	87	9.30	

Within the same population, no significant difference in RVA prevalence was observed between male and female (Table 2).

**Table 2:** RVA positive rates by rRT-PCR screening in male and female outpatients

	Adults				Children			
Gender	No. cases	No. RVA POS	POS rate (%)	χ^2^ (*P*-value)	No. cases	No. RVA POS	POS rate (%)	χ^2^ (*P*-value)
Male	2059	149	7.24	0.01 (0.95)	507	39	7.69	3.41 (0.06)
Female	1907	137	7.18		428	48	11.21	
Total	3966	286	7.21		935	87	9.30	

In adults, the median age in RVA positive cases (46 yrs.) and RVA negative cases (42 yrs.) was not significantly different (Wilcoxon signed-rank test, *P* = 0.10). However, in children, the median age (18 mth.) in RVA positive cases was higher than that in RVA negative cases (15 mth.) (Wilcoxon signed-rank test, *P* < 0.05).

Disparities in RVA prevalence among various age groups were perceived in both adults and children, with the highest prevalence in 45–54 yrs. and 3–5 yrs. group respectively (Tables 3 and 4).

**Table 3:** RVA positive rate in different age groups in adults

Age group (yr.)	No. cases	No. RVA POS	RVA POS rate (%)	χ^2^ (*P*-value)
15–34	1496	98	6.55	2.55 (< 0.05)
35–44	589	36	6.11	
45–54	493	53	10.75	
55–64	733	57	7.78	
≥65	626	39	6.23	
Total	3937	283	7.19

**Table 4:** RVA positive rate in different age groups in children

Age group (yr.)	No. cases	No. RVA POS	RVA POS rate (%)	χ^2^ (*P*-value)
0-6 m	180	11	6.11	9.11 (< 0.05)
7 m–36 m	596	58	9.73	
3-5 yr	102	16	15.69	
6-14 yr	53	2	3.77	
Total	931	87	9.34	

Significant difference in RVA positive rate was observed among different residency groups in pediatric cases, with the highest in local migrants. However, such difference in adult cases was insignificant (Table 5).

**Table 5:** RVA positive rate in different residency groups

	Adults				Children			
Residency group	no. adults	no. RVA pos.	RVA pos. Rate (%)	χ^2^ (*P*-value)	no. children	no. RVA pos.	RVA pos. Rate (%)	χ^2^ (*P*-value)
Local residence	3304	244	7.38	1.86 (0.40)	698	55	7.88	6.70 (< 0.05)
Local migrant^a^	407	29	7.13		151	21	13.91	
Floating population	255	13	5.10		86	11	12.79	
Total	3966	286	7.21		935	87	9.30	

^a^Local migrant is defined as people who have resided in Shanghai for more than 6 months but have not yet obtained permanent residency

Further analysis in pediatric cases showed insignificant differences in RVA prevalence among children living at home, cared in kindergarten, and studying in elementary and middle school (χ^2^ = 2.23, *P* = 0.33) (Table 6).

**Table 6:** RVA positive rate in children with different childcare types

Types of childcare facilities	no. cases	no. RVA pos.	RVA pos. Rate (%)
living at home	783	74	9.45
kindergartens	83	10	12.05
elementary and middle school	48	2	4.17

### Clinical symptoms, epidemiological history and antibiotic administration in RVA cases

Cases positive for RVA and other viral pathogens generally had higher occurrence of nausea and vomiting as the first symptoms accompanying diarrhea in both adults and children (Table 7).

**Table 7:** Occurrence of nausea and vomiting as first symptoms that accompanied diarrhea in cases with different infection status

	Adults				Children			
Cases with different infection status	No. cases	No. vomit accompanied	Rate (%)	χ^2^ (*P*-value)	No. cases	No. vomit accompanied	Rate (%)	χ^2^ (*P*-value)
RVA POS	286	16	5.59	9.07 (< 0.05)	87	5	5.75	(< 0.05)*
POS for other virus	869	59	6.79		188	8	4.26	
Bacteria POS & virus NEG	752	27	3.59		160	0	0.00	
Unknown cause	2059	100	4.86		500	6	1.20	

*Fisher’s exact

On the contrary, fever occurred most frequently in cases infected only by bacteria, and occurred less so in RVA positive cases and cases with other types of viral infection (Table 8).

**Table 8:** Occurrence of fever as one of the first symptoms in cases with different infection status

Cases with different infection status	no. cases	no. cases w/ fever	Rate (%)	χ^2^ (*P*-value)
RVA POS	373	62	16.62	55.15 (< 0.05)
POS for other virus	1057	120	11.35	
Bacteria POS & virus NEG	912	194	21.27	
Unknown cause	2559	312	12.19	

Similarly, the occurrence of abdominal distention and pain is lower in RVA positive cases than that in cases infected with other viruses and bacteria; it was even lower than cases with unknown causes (Tables 9 and 10).

**Table 9:** Occurrence of abdominal distention as one of the first symptoms in cases with different infection status

Cases with different infection status	no. cases	no. cases w/ abdominal distension	Rate (%)	χ^2^ (*P*-value)
RVA POS	373	35	9.38	11.35 (< 0.05)
POS for other virus	1057	149	14.10	
Bacteria POS & virus NEG	912	117	12.83	
Unknown cause	2559	274	10.71

**Table 10:** Occurrence of abdominal pain as one of the first symptoms in cases with different infection status

Cases with different infection status	no. cases	no. cases w/ abdominal pain	Rate (%)	χ^2^ (*P*-value)
RVA POS	373	124	33.24	92.08 (< 0.05)
POS for other virus	1057	389	36.80	
Bacteria POS & virus NEG	912	507	55.59	
Unknown cause	2559	1179	46.07	

When investigating the epidemiological history, it was found that in adult RVA positive cases, more patients recalled the intake of some suspicious food, compared to other cases with a different infection status. However, such pattern was not observed in children (Table 11).

**Table 11:** Percentage of suspicious food intake prior to the onset of diarrhea in cases with different infection status

	Adults				Children			
Cases with different infection status	No. cases	No. cases w/ suspicious food intake	Rate(%)	χ^2^ (*P*-value)	No. cases	No. cases w/ suspicious food intake	Rate(%)	χ^2^ (*P*-value)
RVA POS	286	198	69.23	9.80 (< 0.05)	87	3	3.45	2.42 (0.49)
Other virus POS	869	523	60.18		188	10	5.32	
Bacteria POS & virus NEG	752	450	59.84		160	13	8.13	
Unknown cause	2059	1230	59.74		500	31	6.20	

On the other hand, no significant difference in the rate of pet contact prior to the onset was observed among different groups with difference infection status (Table 12).

**Table 12:** Percentage of pet contact prior to the onset of diarrhea in cases with different infection status

	Adults				Children			
Cases with different infection status	No. cases	No. cases w/ pet contact	Rate(%)	χ^2^ (*P*-value)	No. cases	No. cases w/ pet contact	Rate(%)	χ^2^ (*P*-value)
RVA POS	286	11	3.85	6.27 (0.10)	87	56	64.37	0.32 (0.96)
Other virus POS	869	32	3.68		188	123	65.43	
Bacteria POS & virus NEG	752	23	3.06		160	106	66.25	
Unknown cause	2059	103	5.00		500	320	64.00	

Interestingly, in cases infected by RVA, the rate of self-administration of antibiotics before hospital visit was significantly higher in adults than that in children. Similarly, the same pattern was observed in the rate of antibiotic treatment after the hospital visit (Table 13).

**Table 13:** RVA positive cases with antibiotic administration before and after hospital visit

RVA POS cases	No. cases	No. cases w/ prior antibiotic intake	Rate(%)	χ^2^ (*P*-value)	No. cases treated w/ antibiotics	Rate(%)	χ^2^ (*P*-value)
Adults	286	32	11.19	4.70 (< 0.05)	176	61.54	69.94 (< 0.05)
Children	87	3	3.45		9	10.34	
Total	373	35	9.38		185	49.60	

### Seasonal variation in RVA detection rate

**Fig. 1:**
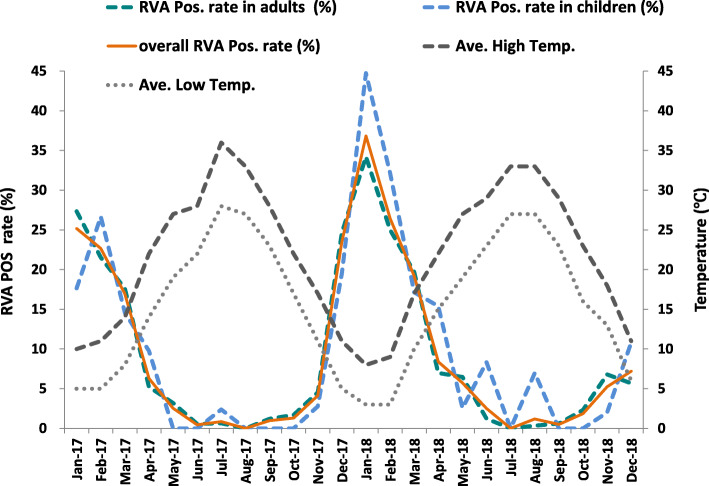
Seasonal prevalence of RVA in both adult and pediatric outpatients

RVA positive detection rate peaked in every winter and reached its highest at 35.53% in Jan 2018 (Fig. 1). On the other hand, RVA positive rate dropped gradually with the rise of atmospheric temperature, and plummeted in summers as shown in Fig. 1.

### RVA genotype diversity

Of 373 RVA positive samples, 284 samples (76.14%) were genotyped successfully. Table 14 shows the percentage composition of various RVA genotypes.

**Table 14:** RVA genotype constitution

RVA genotypes	No. adults	Percentage (%)	No. children	Percentage (%)	Total no. cases	Percentage (%)
G9P[8]	143	65.30	50	76.92	193	67.96
G3P[8]	33	15.07	11	16.92	44	15.49
G2P[4]	32	14.61	3	4.62	35	12.32
G1P[8]	8	3.65	0	0.00	8	2.82
G9P[4]	2	0.91	0	0.00	2	0.70
G10P[15]	0	0.00	1	1.54	1	0.35
G2P[4]+G9P[8]	1	0.46	0	0.00	1	0.35
Total	219	100.00	65	100.00	284	100.00

**Fig. 2:**
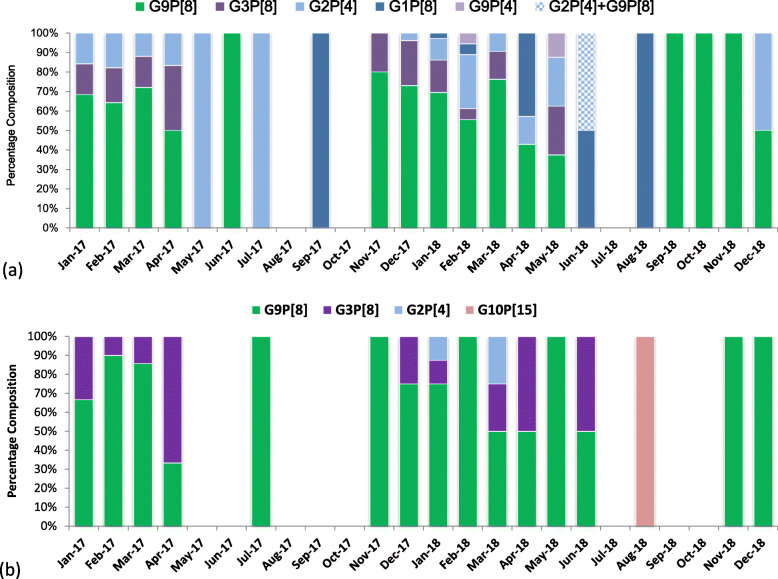
**a** Monthly composition of different RVA genotypes in adults. **b** Monthly composition of different RVA genotypes in children

Monthly composition of different RVA genotypes showed that genotype G9P[8] was the predominant genotype in both adults and children (Fig. 2a, b). Less dominant genotypes such as G3P[8] and G1P[8] were more abundant in seasons with low RVA circulation.

**Fig. 3:**
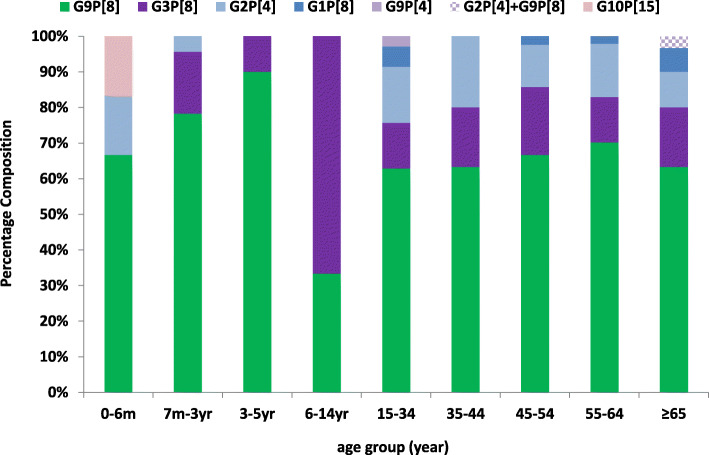
Genotype diversity of RVA in different age groups

Generally speaking, there was a more diverse genotype profile of RVA in adults than that in children, with the highest diversity shown in 15–34 yrs. and ≥ 65 yrs. age group (Fig. 3). Additionally, G9P[8] was detected across all age group.

### Investigation of one G10P[15] case

Genotype G10P[15] was identified in one child with RVA infection. Further investigation of this case revealed the patient to be a migrant girl reaching 5 month of age upon the initial onset of diarrhea. Unfortunately, due to the absence of hospital visit when first symptoms were developed, the original cause of her diarrhea was unknown. Six days after the disease onset, her parents felt the child was well enough to take the first dose of RVA LLR vaccine. However, the mild diarrhea still persisted, which brought about the child’s hospital visit 8 days after vaccine intake. And this is the point where the stool samples were collected and tested. It should be noted that no antibiotics were prescribed or administered prior to or after the hospital visit; and no gastric symptoms were ever present before the development of this ongoing gastroenteritis. Apart from watery diarrhea, no other symptoms were accompanied throughout clinical manifestation.

**Fig. 4:**
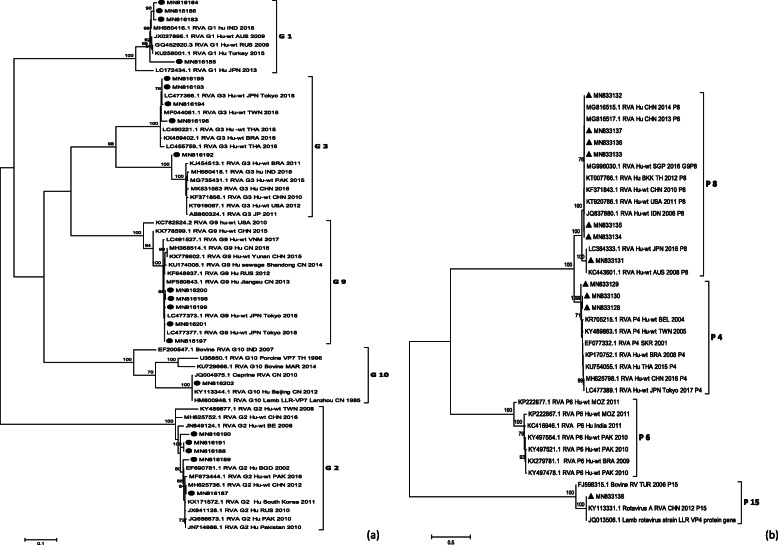
Phylogenetic analysis of RVA (**a**) partial VP7 gene (863 bp) with strains identified in this study shown in black solid circles (**b**) partial VP4 gene (663 bp) with strains identified in this study shown in black solid triangles. The trees were constructed in Mega 6.0 using the maximum likelyhood method with T92 + G + I model for VP7 tree and TN93 + G + I model for VP4 tree. The bootstrap values generated from 1000 replicates are shown at nodes, and only bootstrap values > 70% are shown

In this case, partial VP7 and VP4 gene were sequenced and its lineage was subsequently analyzed. It was found that for both of these above regions, it shared > 99% identity with LLR vaccine (Fig. 4a, b).

## Discussion

Because of the conventional belief that RVA was the leading pathogen in pediatric acute gastroenteritis, its prevalence in adults was often overlooked. In our study, it was shown that RVA prevalence in adult outpatients was as high as 7.21%, albeit being lower than 9.30% in children as expected. Furthermore its prevalence in adults was similar to 7.90% found during 2012–2016 in Shanghai [5]. However the local RVA positive rate in pediatric outpatients was lower than that in pediatric inpatients (13.95%) from our previous study [8], this is probably because RVA is more likely to cause severe cases that result in hospitalization in children [20].

Compared with other regions across China, our rates were lower than the average RVA prevalence (11.2%) from pediatric outpatients in high-income regions, and even much lower than that in low-income regions (40.7%) [6]. It was also much lower than that in countries such as India (36.21% in pediatric cases, and 20.73% in adult cases) [21], Middle East regions and North Africa (19–40%) [22]. On the contrary, the local RVA rate was higher than the median annual rate (6.1%) reported during post vaccine period (2007–2018) in US from CDC’s National Respiratory and Enteric Viruses Surveillance System [23]. These differences might be associated both with the socioeconomic status and the vaccine coverage in the particular region. It should also be noted that before the end of 2018, only LLR vaccine, which contained a single genotype G10P[15] originated from goat, was licensed in China; while multiple multi-valent vaccines with higher efficacy were used in other countries [24].

Interestingly, in recent years in Shanghai, RVA ranked second in causing acute diarrhea in both outpatients and inpatients studies, and in both adults and children, the first being norovirus [5, 8, 11]. Our finding was consistent with the phenomenon observed in US and other developed countries, i.e. the rotavirus vaccine might contribute to the decline of RVA and the rising of other viral pathogens such as norovirus [25]. Moreover, studies conducted in Southern China has confirmed a negative correlation between RVA prevalence and vaccination coverage [26].

Children infected by RVA tend to be older than those not infected by RVA. This was as expected largely due to maternal antibody protection in younger infants [27], RVA vaccination coverage and in some small part, the intake of human milk and dairy fractions, which contained lactoferrin in possessing some antirotaviral activity [28]. Studies in various provinces in China had discovered that RVA prevalence was highest in 1–2 yrs. old population [6]. However, in our study, RVA prevalence was highest in 3–5 yrs. old children, followed by 7–36 mth old children. This discrepancy might be due to a disproportionally smaller sample size in 3–5 yrs. age group in our study. It is believed that while children older than 3 years were often already infected by RVA, and thus causing the elevation of their antibody level in preventing further reinfection [29].

In terms of symptom, higher occurrence of nausea and vomiting, together with fewer occurrences of fever and abdominal discomfort was observed in RVA positive cases, compared with cases infected by bacteria. These milder clinical manifestation in viral diarrhea was consistent with previous findings [5]. It is plausible that specific molecular mechanism of viral infection in gastrointestinal system is one of the contributing factors. For instance, rotaviruses can cause acute severe diarrhea in the absence of substantial intestinal inflammation predominantly due to its ability to inhibit different types of interferons, and thus restricting antiviral and inflammatory functions [30]. As to why suspicious food intake was positively associated with RVA positive adults but not in children, it was probably caused by recall bias in children, whose food history was often answered by their parents, who were quite often reluctant to admit giving child the wrong food. Unfortunately, not all cases could recall the type of food they have taken, and not all mode of transmission were fully explored in the surveillance; more researches need to be conducted to fully confirm the finding.

Nearly 10% of RVA positive cases took antibiotics even before their hospital visit; while around 50% of RVA positive cases were prescribed with antibiotics after their hospital visit. As all of these recruited cases showed only mild symptoms and didn’t require hospitalization, over self-administration and over prescription of antibiotics were implicated in current practice, particularly so in adults. Since overuse of antibiotics is one of the leading causes of antibiotic resistance, and can also cause allergy and adverse effects [31]; results found in this study could offer molecular diagnostic reference for general practitioner in treating acute diarrhea with more discretion, as well as in educational campaign on how to prevent antibiotic abuse in local residents.

RVA displayed a strong seasonality in both adults and children. This was consistent with the finding in the neighboring city in Hangzhou, Zhejiang province, in which a negative correlation between temperature and rotavirus infection rate was established [32]. Similarly higher temperatures were also linked to fewer hospitalizations for rotavirus in both Shanghai and Hong Kong [8, 33]. These phenomenon probably resulted from a more rapid loss of rotavirus infectivity at higher temperatures [34].

Regarding the genotype diversity, commonality in RVA genotype repertoire was shared between adults and children; though child-to-adult transmission was once proposed in adult rotavirus infection [35], more thorough investigation on the dynamic mode of transmission must be conducted before drawing any solid conclusion. In both populations, G9P[8] was the most prevalent genotype, followed by G3P[8] and G2P[4]. Similar genotypes and their prevalence ranking pattern were observed in hospitalized children in Shanghai [8] as well as pediatric outpatients in Beijing [36]. It’s worth noting that G9P[8] is also the most prevalent genotype found in sewage in eastern China [37], which probably served as an environmental reservoir for disease transmission. Since 2008, G9P[8] genotype had replaced its predecessor G3P[8] through genetic evolution in becoming the predominant strain circulating in China [36]. Interestingly, all circulating strains appeared to be a mismatch with G10P[15] identified in LLR vaccine [24]. Studies had revealed that LLR could only offer around 30% protection against the circulating strains [26]. Furthermore, even the newly launched RotaTeq vaccine lacks a G9 genotype in spite of offering a better vaccine efficacy than LLR [9], hence there is still room for optimization in vaccine application.

With one curious case of G10P[15] strain detected in one young infant, such shedding of LLR vaccine was the first reported case in Shanghai. However, similar shedding of vaccine strain was also found in RVA-vaccinated children in Beijing, where fecal shedding occurred as early as post-vaccination day 2 and as late as post-vaccination day 13 and peaked on post-vaccination day 5–10 [38]. In our case, fecal shedding was detected on day 8 after first dose of vaccine, which fell into the range with previous finding [38]. Since LLR was still a live attenuated strain, it has the potential to replicate in human intestine notwithstanding its animal origin [39]. Nevertheless, further information as well as more in-depth research on molecular level was required to determine whether the prolonged mild diarrhea was indeed caused by vaccination.

## Conclusion

RVA displayed a strong seasonality in Shanghai from 2017 to 2018. RVA prevalence was higher in children than in adult outpatients. Clinically, RVA positive cases tend to suffer from milder abdominal discomfort despite having higher occurrence of nausea and vomiting. Furthermore, over self-administration and prescription of antibiotics was indicated in our study. On a molecular level, G9P[8] was the predominant genotype in both children and adults. Moreover, molecular genotyping also facilitated the identification of first case of fecal shedding of LLR vaccine. Overall, it is paramount to continue RVA genotyping through routine surveillance; as it provides an insight into the genetic diversity of RVA both as a baseline and changing trends after the implementation of vaccines. Data obtained from such surveillance system could also be used to evaluate vaccine efficacy in the near future.

## Data Availability

Sequences presented in the result section were deposited in GenBank (accession numbers MN816183–202; MN833128–138). All data involved in this study is available upon reasonable request made to the corresponding authors. GenBank MN816183-MN816202, MN833128-MN833138. MN816183:https://www.ncbi.nlm.nih.gov/nuccore/MN816183.1?report=GenBank MN816184:https://www.ncbi.nlm.nih.gov/nuccore/MN816184.1?report=GenBank MN816185:https://www.ncbi.nlm.nih.gov/nuccore/MN816185.1?report=GenBank MN816186:https://www.ncbi.nlm.nih.gov/nuccore/MN816186.1?report=GenBank MN816187:https://www.ncbi.nlm.nih.gov/nuccore/MN816187.1?report=GenBank MN816188:https://www.ncbi.nlm.nih.gov/nuccore/MN816188.1?report=GenBank MN816189:https://www.ncbi.nlm.nih.gov/nuccore/MN816189.1?report=GenBank MN816190:https://www.ncbi.nlm.nih.gov/nuccore/MN816190.1?report=GenBank MN816191:https://www.ncbi.nlm.nih.gov/nuccore/MN816191.1?report=GenBank MN816192:https://www.ncbi.nlm.nih.gov/nuccore/MN816192.1?report=GenBank MN816193:https://www.ncbi.nlm.nih.gov/nuccore/MN816193.1?report=GenBank MN816194:https://www.ncbi.nlm.nih.gov/nuccore/MN816194.1?report=GenBank MN816195:https://www.ncbi.nlm.nih.gov/nuccore/MN816195.1?report=GenBank MN816196:https://www.ncbi.nlm.nih.gov/nuccore/MN816196.1?report=GenBank MN816197:https://www.ncbi.nlm.nih.gov/nuccore/MN816197.1?report=GenBank MN816198:https://www.ncbi.nlm.nih.gov/nuccore/MN816198.1?report=GenBank MN816199:https://www.ncbi.nlm.nih.gov/nuccore/MN816199.1?report=GenBank MN816200:https://www.ncbi.nlm.nih.gov/nuccore/MN816200.1?report=GenBank MN816201:https://www.ncbi.nlm.nih.gov/nuccore/MN816201.1?report=GenBank MN816202:https://www.ncbi.nlm.nih.gov/nuccore/MN816202.1?report=GenBank MN833128:https://www.ncbi.nlm.nih.gov/nuccore/MN833128.1?report=GenBank MN833129:https://www.ncbi.nlm.nih.gov/nuccore/MN833129.1?report=GenBank MN833130:https://www.ncbi.nlm.nih.gov/nuccore/MN833130.1?report=GenBank MN833131:https://www.ncbi.nlm.nih.gov/nuccore/MN833131.1?report=GenBank MN833132:https://www.ncbi.nlm.nih.gov/nuccore/MN833132.1?report=GenBank MN833133:https://www.ncbi.nlm.nih.gov/nuccore/MN833133.1?report=GenBank MN833134:https://www.ncbi.nlm.nih.gov/nuccore/MN833134.1?report=GenBank MN833135:https://www.ncbi.nlm.nih.gov/nuccore/MN833135.1?report=GenBank MN833136:https://www.ncbi.nlm.nih.gov/nuccore/MN833136.1?report=GenBank MN833137:https://www.ncbi.nlm.nih.gov/nuccore/MN833137.1?report=GenBank MN833138:https://www.ncbi.nlm.nih.gov/nuccore/MN833138.1?report=GenBank

## Abbreviations

RVA: group A rotavirus
rRT-PCR: Real-time Reverse Transcription -Polymerase Chain Reaction
